# Fiber Bragg Grating with Enhanced Cladding Modes Inscribed by Femtosecond Laser and a Phase Mask

**DOI:** 10.3390/s20247004

**Published:** 2020-12-08

**Authors:** Weijia Bao, Shen Liu, Wenjie Feng, Yiping Wang

**Affiliations:** 1 Shenzhen Key Laboratory of Photonic Devices and Sensing Systems for Internet of Things, College of Physics and Optoelectronic Engineering, Shenzhen University, Shenzhen 518060, China; wjbao@szu.edu.cn (W.B.); ypwang@szu.edu.cn (Y.W.); 2 Guangdong and Hong Kong Joint Research Centre for Optical Fibre Sensors, Shenzhen University, Shenzhen 518060, China; 3 Department of Communication Engineering, Nanjing University of Science & Technology, Nanjing 210094, China; wjfengce@njust.edu.cn

**Keywords:** fiber Bragg grating (FBG), fiber cladding mode, femtosecond laser inscription

## Abstract

In this paper, we demonstrate a fiber Bragg grating (FBG) with a wide range and a comb with continuous cladding mode resonances inscribed in non-photosensitive single mode fibers using a femtosecond laser and a phase mask. The FBG is inscribed in the core and cladding, exciting a series of cladding modes in transmission. The birefringence induced by this FBG structure offers significant polarization-dependence for cladding modes, thus allowing the vector fiber twist to be perceived. By measuring the peak-to-peak differential intensity of orthogonally polarized cladding mode resonances, the proposed sensor presents totally opposite intensity response in the anticlockwise direction for the torsion angle ranging from −45° to 45°. The cladding mode comb approximately covers wavelengths over the O-, E-, S-, and C-bands in transmission. The cutoff cladding mode of air can be observed in the spectrum. Thus, the sensible refractive index range is estimated to be from 1.00 to 1.44. Temperature responsivity of the grating is also characterized. The proposed device potentially provides new solutions to the various challenges of physical vector and bio-chemical parameters sensing.

## 1. Introduction

Fiber cladding mode-based sensors have attracted widespread attention due to their unique sensing properties. Various bio-chemical or physical parameters, such as liquid refractive index (RI), bending, strain, and temperature, can effectively modify the cladding mode characteristics (including central wavelength and intensity). Generally, a fiber Bragg grating (FBG) involves a core mode and a comb with slight cladding modes that is induced by the coupling between the forward-propagating core mode and backward-propagating modes along the cladding in the transmission spectrum. To magnify this effect and further utilize cladding modes for measurement, various sensing configurations have been investigated [1,2,3,4]. Core-mismatch splicing is a conventional and simple method for cladding mode excitation, and the related sensor structures are numerous in their variety [5,6,7,8]. Long period grating (LPG) is a classical device for coupling from the core mode to co-propagating cladding modes [9,10], and is an effective converter for the core-to-cladding mode. However, LPG can only couple the core mode to low-order cladding modes, and the structure is not compact. Tilted fiber Bragg grating (TFBG) has better performance, which is attributed to its unique mode coupling and spectral properties [11,12,13,14,15], whereas the inscription of the initial TFBG devices has a high requirement of fiber photosensitivity. Thus, TFBG in non-photosensitive fiber inscribed by a femtosecond laser is a growing concern [16,17,18,19]. Alternatively, highly localized FBGs induced by a femtosecond laser can also achieve strong cladding mode resonance excitation [20,21,22]. However, these grating inscriptions produced using femtosecond laser usually require precise alignment and motion control.

In addition to the enhancement of the cladding mode, the optimization of cladding mode sensing properties is also a crucial research direction. The physical and biochemical sensing capacity can be improved by post-processing or modified fabrication, as noted in the references mentioned above [14,15,16,17,18,19]. For instance, the polarization dependence of cladding modes responds readily to the vector physical parameter that involves magnitude and direction. Two orthogonal polarizations of cladding modes respond to both orientation and amplitude of these applied vector parameters, such as FBG written in the polarization-maintained fiber for vibration [23], TFBG with excessive angle of 81° for twist [24,25], re-grown TFBG with split cladding mode for bending [26], and TFBG with metallic oxide coating for twist [27]. In these studies, the researchers fully utilize the strain response difference of two orthogonal polarized resonances (including p- and s-polarization) to achieve vector measurements. In addition, the wavelength range and amount of cladding modes are also highly relevant, particularly for RI measurement. These can be modified by designed grating inscription, such as off-axis TFBG fabricated by femtosecond laser line-by-line technique [19], multi-angle TFBG fabricated by a scanned phase-mask technique [28], moiré TFBG fabricated by using a direct phased mask with two-stage UV exposure [29], and FBG fabricated by a small number of femtosecond laser pulse exposures [30]. Therefore, the coupling, polarization dependence, and wavelength range are equally important in a cladding mode-based sensor.

In this work, a FBG with a wide range and comb with continuous cladding mode resonances is inscribed in non-photosensitive single mode fiber using a femtosecond laser and phase mask. In particular, the refractive index modification is introduced in the both of the core and cladding due to the nonlinear light-material absorption induced by high-intensity laser exposure. We obtain the comb of cladding mode resonances in the transmission spectrum, which shows a significant birefringence effect (cladding modes split into two distinct polarized states). The FBG length can effectively influence the spectral characteristics (including wavelength range and transmission depth). Thus, the cladding modes of the proposed FBG are characterized as highly efficient coupling, significant polarization dependence, and wide wavelength range. Based on these characteristics, the proposed sensor enables vector twist measurement and wide-range RI measurement.

## 2. Fabrication and Characterization

**Figure 1:**
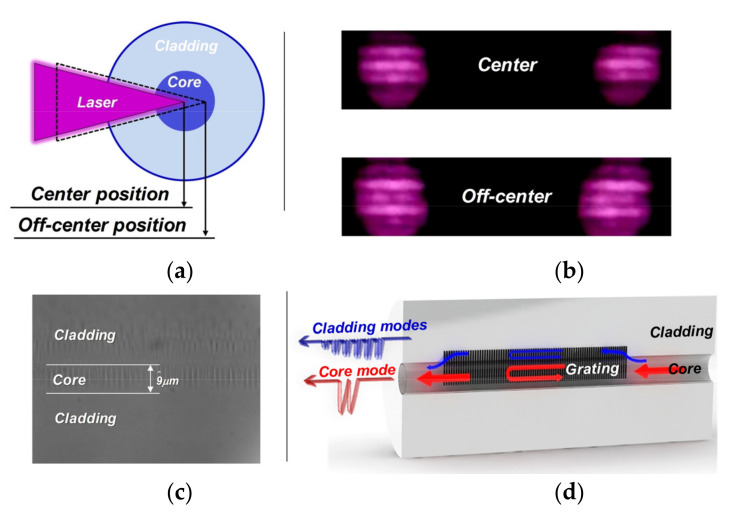
(**a**) Schematic of the focused point inside the fiber; (**b**) diffraction patterns of the laser after passing through the fiber core for focused beam and defocused beam; (**c**) longitudinal photomicrograph of grating; (**d**) schematic of grating.

The investigated FBG was inscribed in standard Nufern SMF-28 fiber using the classic phase mask technique. The femtosecond laser used was generated by a Ti:sapphire regenerative amplifier system (Spectra-Physics, Solstice, Santa Clara, CA, USA), which emits a pulse width of 100 fs and output pulse energy of 4 mJ with linearly polarized light at a central wavelength of 800 nm. The pulse energy can be adjusted via rotation of a half-wave plate followed by a Glan polarizer. The laser beam was focused into the fiber by a cylindrical lens with a 15.5 mm focal distance that was mounted on a two-dimensional stage with a resolution of 10 μm for adjusting the position. To observe the fiber position, we set up a microscope over the inscription stage. A uniform phase mask (Ibsen Photonics, Farum, Denmark) with zero-order diffraction efficiency below 4% and grating pitch of 2120 nm was positioned between the fiber and lens. The mask was positioned approximately 300 μm from the fiber for high RI modification efficiency to ensure the narrowest focused laser line and highest peak intensity in the focus [31]. In particular, the laser was not focused in the center of the fiber but in the interface between the core and cladding, as shown in Figure 1a. The focused position inside the fiber can be estimated via observing the laser diffraction pattern after passing through the fiber core, see Figure 1b. When the laser beam focus position is in the core center, the middle zero-order diffraction fringe is strong. However, the zero-order diffraction fringe will gradually weaken when the laser beam focus position is off-center. Therefore, we can use the diffraction pattern as the criterion for the focus position. High intensity femtosecond laser modification is a process of energy deposition in materials, which can result in complex modification (strong RI change and material damage) and significant propagation loss. The laser exposure parameters must be appropriate for grating inscription. With a high pulse energy of 0.8 mJ and exposure duration of 30 s, a highly RI-modified region can be formed in both the core and cladding [20,22], as shown in the photomicrograph in Figure 1c. The strong RI modification enhances the coupling of the core mode to the cladding modes. A special FBG with a wide range and comb with continuous cladding mode resonances (defined as a cladding mode FBG, CMFBG) can be achieved with this modification. Although transmission achieves a similar result to that of a TFBG [11] or an eccentric point by point (PbP) grating [32,33], it is superior to these two. Exciting cladding modes without a specialized/tilted mask is important because a tilted mask costs significantly more than a uniform mask. In addition, the PbP technique requires careful alignment and precision.

The Bragg wavelength is well-known to be:

*λ* = 2*n_eff_Λ*/*m* (1)

where *n_eff_* is the effective refractive index of the core mode, *Λ* is the Bragg grating period, and *m* is the order of grating. As shown in Figure 1c, due to the grating in the core and cladding, the mode coupling between the forward propagating and counter-propagating core modes contributes the core mode resonance. In addition, more coupling between the core mode and definite cladding modes characterized by their radial and azimuthal mode numbers contributes a multitude of cladding mode resonances [21]. For the *i*-order cladding mode, resonance wavelengths can be expressed as:

*λ* = (*n_eff_* + *n_eff, i_*)*Λ*/*m* (2)

The cladding mode resonance highly depends on the RI modification region that governs the coupling efficiency of cladding modes.

**Figure 2:**
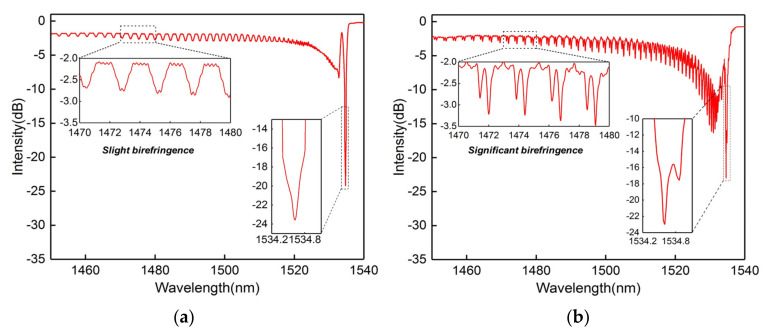
Transmission spectrum of the fiber Bragg grating (FBG) without (**a**) and with (**b**) enhanced cladding modes (inset: magnified spectrum of cladding modes and core mode from 1470 to 1480 nm).

The grating region will break the cylindrical symmetry of the fiber and predictably result in the birefringence effect. A common FBG was inscribed with the normal focused laser condition to verify the focused laser exposure position effect. As shown by the spectral response in Figure 2a, the cladding mode resonances are obtained in the short wavelength side. The high intensity laser pulse introduces a slight birefringence in the fiber material, so the cladding mode peaks are slightly split into two peaks; see inset of Figure 2a. However, the localized grating-induced birefringence offers a strong polarization-dependence in this work, and the core mode and cladding modes are significantly split into two peaks. These two core mode resonances are separated by 270 pm, corresponding to a birefringence value of approximately 2.6 × 10^−4^. The comparable cladding mode spectral response can be observed in the high-order cladding mode [34] and the large angle TFBG [25]. In contrast to the common FBG, the cladding modes are significantly improved, particularly for the low-order modes, as shown by the spectral response in Figure 2b. Each cladding mode has a one-to-one related effective RI value and will vanish when the surround RI is close to or beyond that value [11,12]. Thus, the spectral range is an important property for cladding mode-based sensors, and the broader-range cladding mode comb has a wider measurement range.

**Figure 3:**
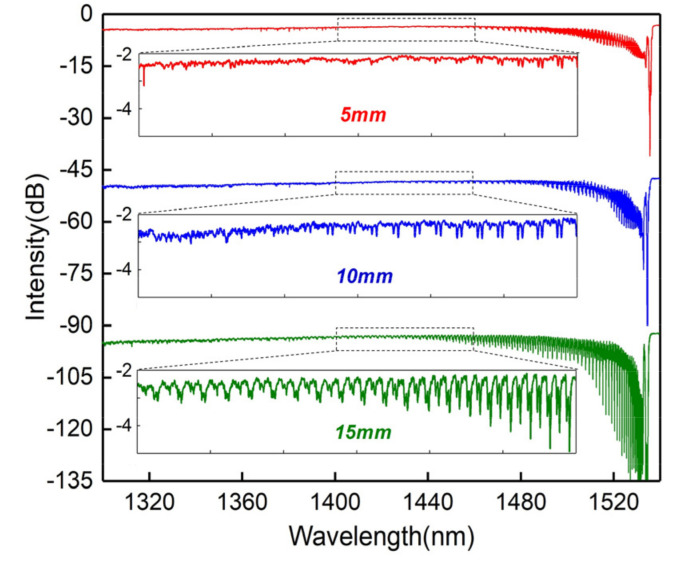
Transmissions of a cladding mode FBG (CMFBG) versus different grating lengths, inset: magnified spectrum from 1400 to 1460 nm.

To further improve the cladding mode range, we studied the effect of the grating length on the spectral response. The grating length was controlled by the phase mask scanning method. Generally, a longer grating length with the same RI modification provides stronger reflectivity, and weak cladding modes can be further enhanced. Figure 3 shows the evolution of transmitted spectra (grating lengths are 5, 10, and 15 mm). According to the results, the grating length effectively enhances the core-to-cladding mode coupling, and the peak-to-peak amplitude and spectral range of the cladding mode resonances are gradually improved with increasing grating length.

**Figure 4:**
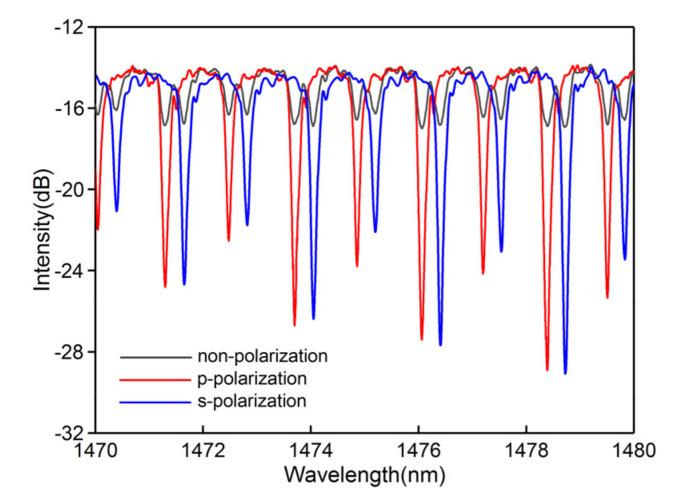
Transmission of cladding mode resonances at three different polarization states.

To characterize the polarization dependence of the CMFBG, an in-line polarizer and a polarization controller (PC) were placed in front of the CMFBG. The polarization state of the light shone on the CMFBG can be changed by the PC. We used a 15 mm CMFBG as an example. Figure 4 shows the magnified transmission spectrum of the low-order cladding modes from 1470 to 1480 nm. The attenuations of cladding mode doublets are almost the same when the light is orthogonally polarized. Using the appropriate polarized light, the doublets present a suppressed or strengthened trend. Here, one of the peaks reaches maximum transmission intensity, whereas another almost disappears in the transmission. The polarization dependence benefits from strong coupling between the core mode and azimuthal cladding modes, which is induced by the asymmetric RI modification [11,21].

## 3. Experiments and Discussions

**Figure 5:**
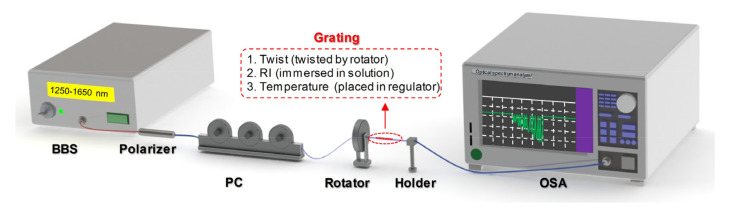
Setup scheme for sensing experiments.

Taking advantage of the proposed CMFBG, multiple sensing measurements can be realized. In this section, we characterize the vector twist, RI, and temperature responsiveness of the CMFBG, and discuss the experimental results. We used a similar setup as that described in Section 2 for the sensing measurement, as shown in the scheme in Figure 5. The transmission spectra were recorded via an optical spectrum analyzer (OSA, Yokagawa AQ6370D, Tokyo, Japan) and a broadband light source (BBS) with a spectrum range of 1250–1650 nm. The unpolarized input light from the BBS can be precisely controlled by the polarizer and the PC. The twist angle of the grating was applied by a rotator connected to one end of the grating (the other end was fixed). For three different measurements, the grating sample was twisted by a rotator (twist), immersed in solution (RI), and placed in a temperature regulator (temperature).

### 3.1. Vector Twist

**Figure 6:**
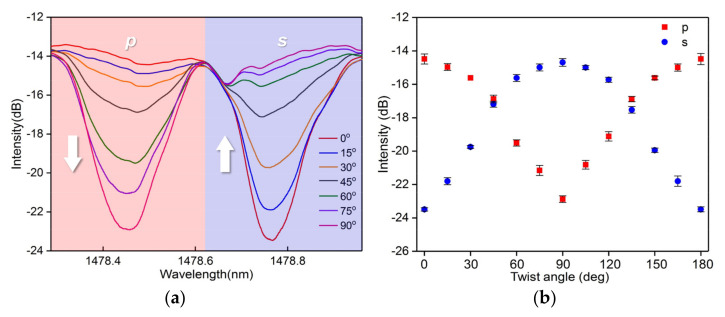
(**a**) spectral response to the fiber twist around 1478.6 nm; (**b**) intensity variation of p- and s-polarized states.

We used the same setup as that used for polarization dependence measurement for vector twist measurement. The vector twist involves the angle magnitude and the rotation direction. To measure the vector twist, we need to measure the twist angle and distinguish the rotation direction. The grating planes of the CMFBG break the azimuth symmetry of the fiber, as discussed above. The polarization state of the input electric field vector is dependent on the angle between the field vector and grating planes. Assuming that the initial field vector is parallel to the grating planes (i.e., p-polarized), it will change as a result of fiber twisting because the angle between the field and planes is changed. Thus, two orthogonal polarization states (p- and s-polarization) of the corresponding excited cladding mode can be modified by the fiber twist. The default input light was s-polarized. We selected a cladding mode of around 1478.6 nm as the sensing reference. As shown by the spectral response in Figure 6a,b, p-polarization presents an opposite evolution compared to s-polarization with an increase in the fiber twist angle. This is crucial for recognition of the twist direction (clockwise or anticlockwise). Here, the wavelength shows a slight shift during the measurement, which is attributed to the fiber twist-induced RI change and the non-ideal alignment of the rotator.

**Figure 7:**
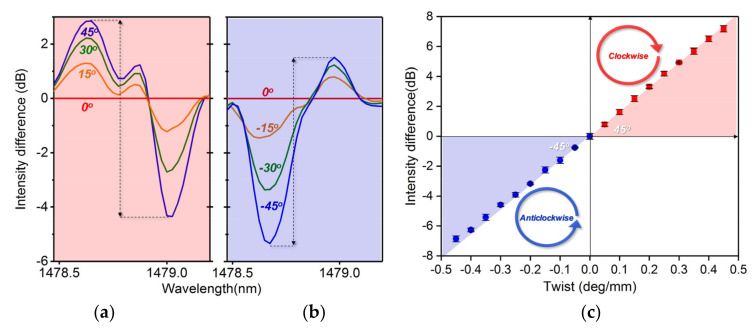
Differential spectrum response to the fiber twist around 1478.6 nm: (**a**) p-polarization; (**b**) s-polarization; (**c**) peak-to-peak intensity response to fiber twist in different directions.

Notably, the magnitudes of the variation of the two polarizations due to the angle change are different. They are more sensitive when the orientation of the grating planes is close to the field vector, and the intensity responses are not linear; see Figure 6b. However, we can observe that the intensity variations of the two polarizations for each step increase in the angle are approximately equal from the red and blue zone in Figure 6a (corresponding to p- and s-polarization, respectively). Therefore, we set the case that p-polarization is equal to s-polarization as the standard state, and applied the difference disposal to the spectral response; see Figure 7a,b. The peak-to-peak intensity of the differential spectrum between the p- and s-polarized resonances is zero at a twist angle of 0°. The twisted fiber length is 100 mm, and the sensitivity is dependent on the fiber length. So the sensor presents a positive variation trend with a great linear sensitivity of 16.2 dB/deg∙mm when increasing the twist angle from 0° to 45° (clockwise), while it presents a negative variation trend with a great linear sensitivity of −15.3 dB/deg∙mm when decreasing the twist angle from 0° to −45° (anticlockwise), as shown in Figure 7c. This intensity response characteristic provides a direction recognition mechanism for the vector twist (positive variation for clockwise, negative variation for anticlockwise).

### 3.2. Refractive Index

We now demonstrate the sensing properties of the CMFBG. The CMFBG can couple the core mode to the cladding modes, which hold a core mode resonance and then introduce additional multi cladding mode resonances in transmission. Each cladding mode has a one-to-one related effective RI value based on the phase matching condition. Furthermore, it becomes leaky when the surround RI is close to or beyond that value, yielding a highly surround RI sensitivity.

**Figure 8:**
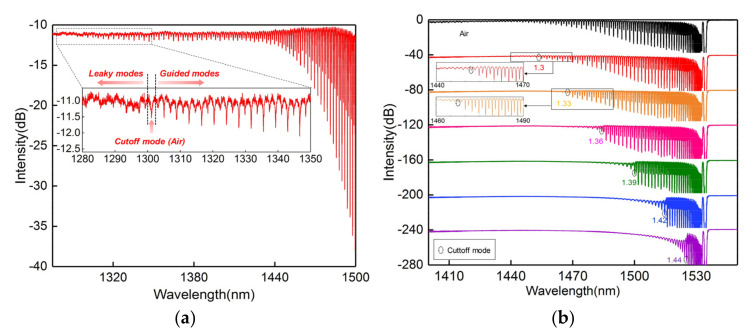
(**a**) Transmission in the air (effective refractive index (RI) ~ 1000); (**b**) spectral responses to different RI solutions; inset: magnified spectrum around the cutoff mode.

**Figure 9:**
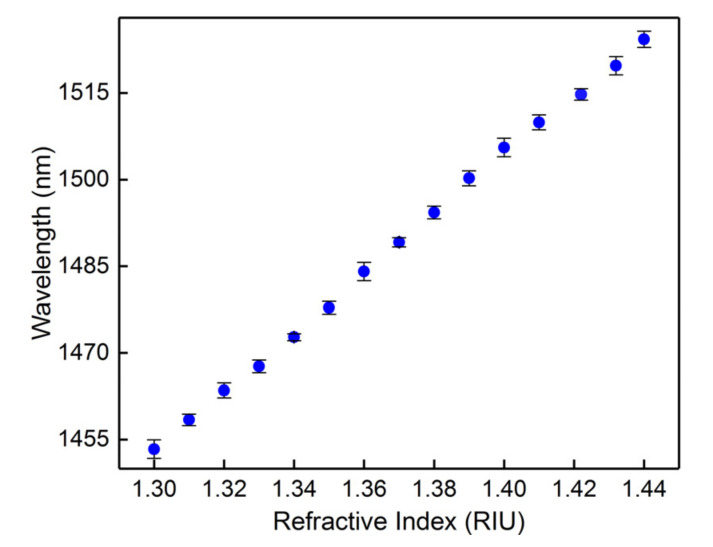
Effective RI sensitivity of the cutoff mode.

We used a 15 mm CMFBG for the surround RI measurement; the cladding mode comb in the short wavelength side is shown in Figure 8a. The input light is s-polarized to facilitate the spectral analysis. We can observe that the cladding mode is continuous and the cutoff is around 1301 nm from the insert because the effective RI of the cladding mode here is estimated to be 1.0 (effective RI of air). The cutoff mode is the boundary between the guided modes and leaky modes. Thus, a portion of the cladding modes located at the short wavelength side of the cutoff mode vanish as the RI increases, and are transformed into leaky modes. A series of RI-matching fluids ranging from 1.33 to 1.44 were prepared as samples. For consecutive tests, the entire grating region was immersed in fluid, and cleaned with alcohol after each measurement. The sensing spectrum was restored to its original state after cleaning. The spectral responses of the CMFBG produced to surround the RI are demonstrated in Figure 8b. The intensities of the guided modes were not changed with the external RI, and maintain their original spectral characteristic. However, the transformed leaky modes will weaken relative to their original state, or even vanish, as shown by the spectral evolutions in Figure 8b. Therefore, the boundary (cutoff mode) between the guided modes and leaky modes, which gradually shifts to the long wavelength side, is a criterion for RI measurement. We plotted the wavelength for the cutoff mode as a function of the surround RI with a linear sensitivity of 510.95 nm/RIU, as shown in Figure 9.

### 3.3. Temperature

**Figure 10:**
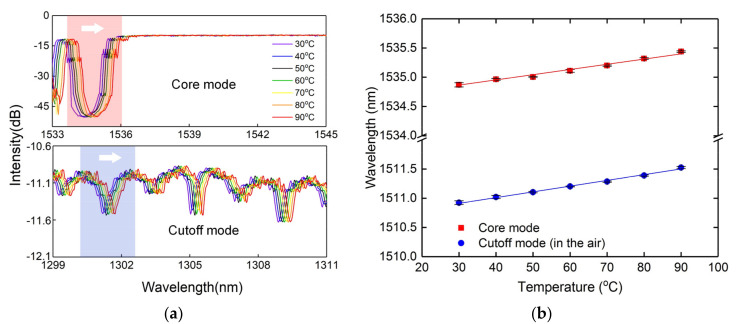
(**a**) Spectral responses to temperature of the core mode and cutoff mode; (**b**) Temperature sensitivities of the core mode and cutoff mode.

The thermal property of the CMFBG was also characterized. To facilitate the analysis, the default input light in temperature measurement is still s-polarized. The CMFBG was placed inside a temperature box with a resolution of 0.1 °C. The temperature increased with a step of 10 °C, and was maintained for five minutes to ensure the evenness of the temperature in the box. As the temperature increased, the overall spectrum responses showed wavelength shifting without significant fluctuation in intensity, as the spectral responses of core mode and cutoff mode in Figure 10a. We plotted the wavelength of the core mode and cutoff mode as a function of temperature with a sensitivity of 9.3 pm/°C and 8.7 pm/°C, as shown in Figure 10b. Thus, the proposed CMFBG has the potential for multi-parameter sensing.

## 4. Conclusions

In conclusion, a CMFBG was inscribed in the fiber core and cladding by a femtosecond laser and phase mask. The CMFBG held a core mode resonance and cladding mode comb in transmission, and showed significant polarization dependence. The range of the cladding mode comb could be broadened by extending the FBG length. The wide comb ranging from 1301 to around 1530 nm could be realized by a 15 mm CMFBG. The CMFBG showed an excellent performance in fiber twist, and thus can effectively distinguish twist direction. Moreover, it showed high RI sensitivity with a measurement range from 1.0 to 1.44 and good thermal stability. It has many advantages, such as easy inscription, high core-to-cladding mode efficiency, significant polarization dependence, and a wide-range cladding mode spectrum, making it a potential candidate for multi-parameter measurement.

## References

[1] Eggleton B., Westbrook P., White C.A., Kerbage C., Windeler R.S., Burdge G.L. (2000). Cladding-Mode-resonances in air-silica microstructure optical fibers. J. Light. Technol..

[2] Pang F.F., Liang W.B., Xiang W.C., Chen N., Zeng X.L., Chen Z.Y., Wang T.Y. (2009). Temperature-insensitivity bending sensor based on cladding-mode resonance of special optical fiber. IEEE Photon. Technol. Lett..

[3] Bao W.J., Qiao X.G., Rong Q.Z. (2019). Fiber-optic vector accelerometer using orthogonal Bragg grating inscription over innermost cladding of a multi-clad fiber. J. Light. Technol..

[4] Ascorbe J., Corres J., Matias I., Arregui F. (2016). High sensitivity humidity sensor based on cladding-etched optical fiber and lossy mode resonances. Sens. Actuators B Chem..

[5] Zhou W.J., Zhou Y., Dong X.Y., Shao L.Y., Cheng J., Albert J. (2012). Fiber-optic curvature sensor based on cladding-mode Bragg grating excited by fiber multimode interferometer. IEEE Photon. J..

[6] Ma Y., Qiao X.G., Guo T., Wang R.H., Zhang J., Weng Y.Y., Rong Q.Z., Hu M.L., Feng Z.Y. (2012). Reflective fiber-optic refractometer based on a thin-core fiber tailored Bragg grating reflection. Opt. Lett..

[7] Rong Q.Z., Qiao X.G., Zhang J., Wang R.H., Hu M.L., Feng Z.Y. (2012). Simultaneous measurement for displacement and temperature using fiber Bragg grating cladding mode based on core diameter mismatch. J. Light. Technol..

[8] Bao W.J., Hu N.F., Qiao X.G., Rong Q.Z., Wang R.H., Yang H.Z., Yang T.T., Sun A. (2016). High-temperature properties of a thin-core fiber MZI with an induced refractive index modification. IEEE Photon. Technol. Lett..

[9] Han M., Guo F.W., Lu Y.F. (2010). Optical fiber refractometer based on cladding-mode Bragg grating. Opt. Lett..

[10] Enríquez D.A.C., Cruz A.R., Giraldi M.T.M.R. (2012). Hybrid FBG–LPG sensor for surrounding refractive index and temperature simultaneous discrimination. Opt. Laser Technol..

[11] Albert J., Shao L.Y., Caucheteur C. (2013). Tilted fiber Bragg grating sensors. Laser Photon. Rev..

[12] Guo T., Liu F., Guan B.O., Albert J. (2016). Tilted fiber grating mechanical and biochemical sensors. Opt. Laser Technol..

[13] Guo T., Liu F., Shao L.Y. (2018). Tilted fiber Bragg grating sensors. J. Appl. Sci..

[14] Zhang Z.C., Guo T., Guan B.O. (2019). Reflective fiber-optic refractometer using broadband cladding mode coupling mediated by a tilted fiber Bragg grating and an in-fiber mirror. J. Light. Technol..

[15] Caucheteur C., Guo T., Liu F., Guan B.O., Albert J. (2016). Ultrasensitive plasmonic sensing in air using optical fibre spectral combs. Nat. Commun..

[16] Chen C., Yu Y.S., Yang R., Wang C., Guo J.C., Xue Y., Chen Q.D., Sun H.B. (2013). Reflective optical fiber sensors based on tilted fiber Bragg gratings fabricated with femtosecond laser. J. Light. Technol..

[17] Wang R.Z., Si J.H., Chen T., Yan L.H., Cao H.J., Pham X.T., Hou X. (2017). Fabrication of high-temperature tilted fiber Bragg gratings using a femtosecond laser. Opt. Express.

[18] Ioannou A., Theodosiou A., Caucheteur C., Kalli K. (2017). Direct writing of plane-by-plane tilted fiber Bragg gratings using a femtosecond laser. Opt. Lett..

[19] Pham X.T., Si J.H., Chen T., Qin F.H., Hou X. (2019). Wide range refractive index measurement based on off-axis tilted fiber Bragg gratings fabricated using femtosecond laser. J. Light. Technol..

[20] Grobnic D., Smelser C.W., Mihailov S., Walker R.B., Lu P. (2004). Fiber Bragg gratings with suppressed cladding modes made in SMF-28 with a femtosecond IR laser and a phase mask. IEEE Photon. Technol. Lett..

[21] Thomas J., Jovanovic N., Becker R.G., Marshall G.D., Withford M.J., Tünnermann A., Nolte S., Steel M.J. (2011). Cladding mode coupling in highly localized fiber Bragg gratings: Modal properties and transmission spectra. Opt. Express.

[22] Bao W.J., Qiao X.G., Rong Q.Z., Hu N.F., Yang H.Z., Feng Z.Y., Hu M.L. (2015). Sensing characteristics for a fiber Bragg grating inscribed over a fiber core and cladding. IEEE Photon. Technol. Lett..

[23] Guo T., Shang L.B., Liu F., Wu C., Albert J. (2013). Polarization-maintaining fiber-optic-grating vector vibroscope. Opt. Lett..

[24] Zhou K.M., Zhang L., Chen X.F., Bennion I. (2006). Optic sensors of high refractive-index responsivity and low thermal cross sensitivity that use fiber Bragg gratings of >80° tilted structures. Opt. Lett..

[25] Yan Z.J., Wang H.S., Wang C.L., Sun Z.Y., Yin G.L., Zhou K.M., Wang Y.S., Zhao W., Zhang L. (2016). Theoretical and experimental analysis of excessively tilted fiber gratings. Opt. Express.

[26] Shao L.Y., Xiong L.Y., Chen C.K., Laronche A., Albert J. (2010). Directional bend sensor based on re-grown tilted fiber Bragg grating. J. Light. Technol..

[27] Wang R.L., Li Z.H., Chen X., Hu N., Xiao Y.G., Li K.W., Guo T. (2020). Mode splitting in ITO-nanocoated tilted fiber Bragg gratings for vector twist measurement. J. Light. Technol..

[28] Chen X.Y., Xu J., Zhang X.J., Guo T., Guan B.O. (2017). Wide range refractive index measurement using a multi-angle tilted fiber Bragg grating. IEEE Photon. Technol. Lett..

[29] Wang T., Liu K., Jiang J.F., Xue M., Chang P.X., Liu T. (2017). Temperature-insensitive refractive index sensor based on tilted moiré FBG with high resolution. Opt. Express.

[30] Abdukerim N., Grobnic D., Hnatovsky C., Mihailov S.J. (2020). High-temperature stable fiber Bragg gratings with ultrastrong cladding modes written using the phase mask technique and an infrared femtosecond laser. Opt. Lett..

[31] Abdukerim N., Grobnic D., Lausten R., Hnatovsky C., Mihailov S.J. (2019). Complex diffraction and dispersion effects in femtosecond laser writing of fiber Bragg gratings using the phase mask technique. Opt. Express.

[32] González-Vila Á., Ioannou A., Loyez M., Debliquy M., Lahem D., Caucheteur C. (2018). Surface plasmon resonance sensing in gaseous media with optical fiber gratings. Opt. Express.

[33] Chah K., Kinet D., Caucheteur C. (2016). Negative axial strain sensitivity in goldcoated eccentric fiber Bragg gratings. Sci. Rep..

[34] Theodosiou A., Kalli K., Caucheteur C. (2018). Higher-order cladding mode excitation of femtosecond-laser-inscribed tilted FBGs. Opt. Lett..

